# Microbiome Impact on Amyloidogenesis

**DOI:** 10.3389/fmolb.2022.926702

**Published:** 2022-06-16

**Authors:** Jofre Seira Curto, Amat Surroca Lopez, Maria Casals Sanchez, Iva Tic, Maria Rosario Fernandez Gallegos, Natalia Sanchez de Groot

**Affiliations:** Self-organization in Biological Systems Lab, Department of Biochemistry and Molecular Biology, Biosciences Faculty, Universitat Autònoma de Barcelona, Cerdanyola del Vallès, Spain

**Keywords:** microbiome, amyloid, prion, bacteria, probiotic, neurodegenerative disease, gut

## Abstract

Our life is closely linked to microorganisms, either through a parasitic or symbiotic relationship. The microbiome contains more than 1,000 different bacterial species and outnumbers human genes by 150 times. Worryingly, during the last 10 years, it has been observed a relationship between alterations in microbiota and neurodegeneration. Several publications support the hypothesis that amyloid structures formed by microorganisms may trigger host proteins aggregation. In this review, we collect pieces of evidence supporting that the crosstalk between human and microbiota amyloid proteins could be feasible and, probably, a more common event than expected before. The combination of their outnumbers, the long periods of time that stay in our bodies, and the widespread presence of amyloid proteins in the bacteria Domain outline a worrying scenario. However, the identification of the exact microorganisms and the mechanisms through with they can influence human disease also opens the door to developing a new and diverse set of therapeutic strategies.

## Introduction

**FIGURE 1:**
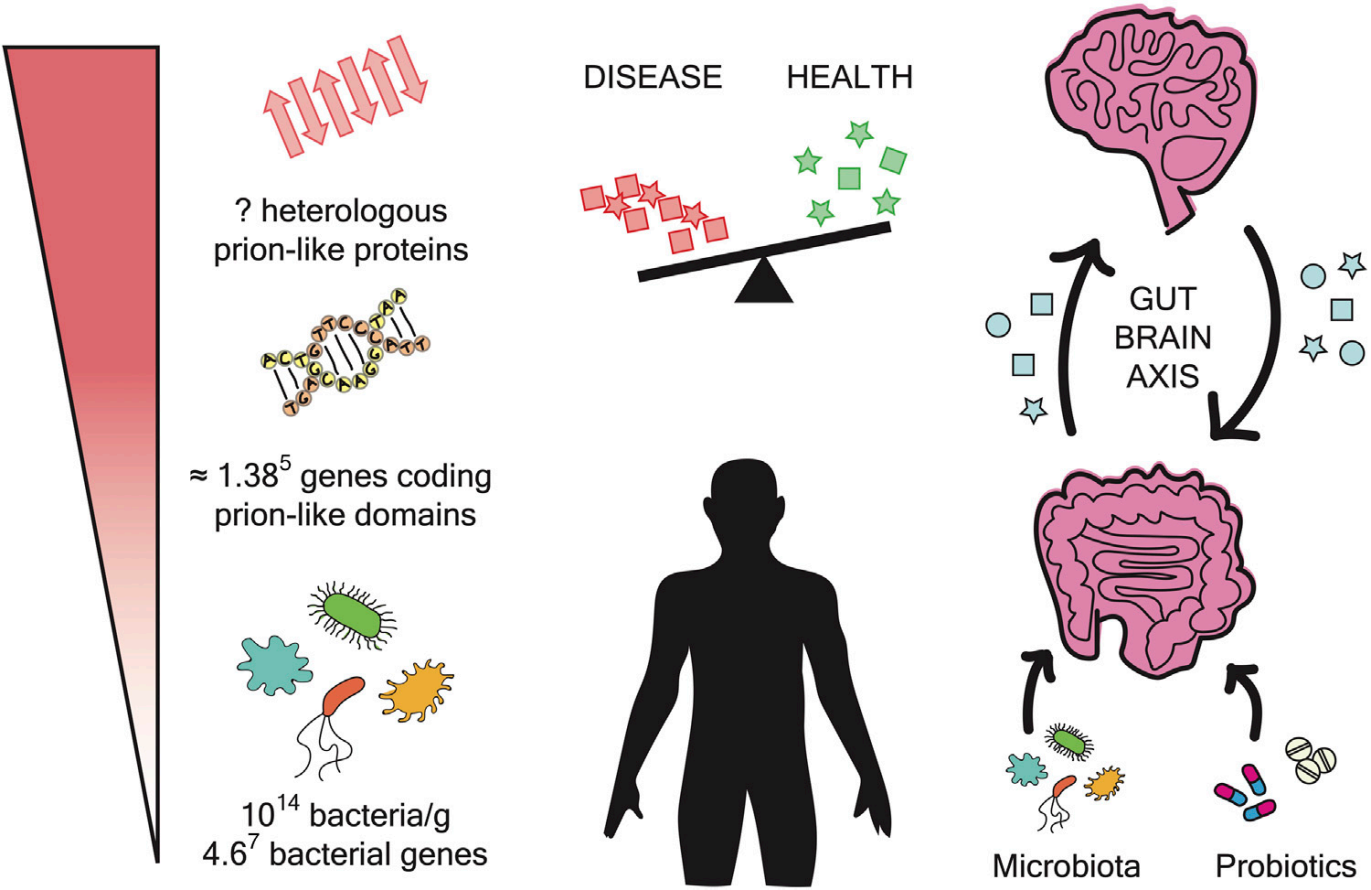
Microbiome impact on amyloidogenesis. The gut microbiome is composed of approximately 10^14^ bacteria and contains 4.6^7^ bacterial genes. The prion prediction tools have measured approximately 0.3% prions per genome. In the microbiome, this can result in approximately 1.38^5^ genes coding for prion-like domains. The expression of these genes can produce a large number of polypeptide sequences with the potential to form amyloid fibrils. These aggregates may have the ability to interfere with and cross-seed human proteins. The gut and the brain are interconnected by a bidirectional axis. In addition, it has been reported that changes in microbiota composition are related to neurodegenerative diseases. Overall, the administration of probiotics could be a potential therapeutic strategy to treat these disorders.

In 2009 the composition of the human microbiome (∼10^13^ microbial cells) was published (Figure 1). The largest microbial community resides in the gut, where microbial cells outnumber human cells by about 10:1 and their genes by about 100:1(NIH Human Microbiome Project—HMRGD, 2022; Qin et al., 2010). Most of these cells are bacteria, and fungi just represent between 1% and 2% of the biomass (Iliev and Cadwell, 2021). According to these estimations, the microbiota has been designated as the largest “diffuse organ system” in the human body. But more important than its size is its metabolic activity, which is larger than the liver and supports many vital processes (Hill and Lukiw, 2015). Gut microbiota contributes to carbohydrate fermentation and absorption, competes with pathogens, metabolizes and neutralizes dietary carcinogens, and takes part in innate immunity supporting infection and disease resistance (Bhattacharjee and Lukiw, 2013). Thanks to the gut-brain bidirectional communication system, it also influences neuroinflammation, neuromodulation, and neurotransmission (Zhao et al., 2015). In parallel, microorganisms can also colonize our bodies through parasitic and pathogenic mechanisms (Hill et al., 2014).

Microbiota composition can be affected by several factors such as age, gender, environment, diet, or medical treatments (Hill et al., 2014; Miller et al., 2021). These compositional changes can lead to dysbiosis and the consequent disturbance of human health. As follows, microbiota imbalance has been associated with autoimmune and inflammatory disorders (inflammatory bowel disease, asthma, allergies) and with the acceleration of chronic diseases such as cardiovascular disease, obesity, diabetes or cancer (Wilkins et al., 2019; Miller et al., 2021; Liu et al., 2022). Also, several recent studies have identified microbiota dysbiosis in patients affected by different neurodegenerative diseases pointing to a possible connection between the gut microbiota and the origin of neurological disorders (Figure 1) (Rogers et al., 2016; Friedland and Chapman, 2017).

The accumulation of amyloid fibrils in the brain is a common hallmark associated with neurodegenerative diseases, such as Alzheimer’s or Parkinson’s. Despite that in each disorder the aggregates are composed of different proteins, the biophysical properties that define the amyloid structure are the same (Tjernberg et al., 2016; Walker et al., 2016). Interestingly, amyloid fibrils are highly resistant and organized and their conformation can be transmitted, in a prion-like manner, to other proteins even without sequential similarities (Table 1) (Zhou et al., 2012; Kosolapova et al., 2020; Subedi et al., 2022). These special properties have been exploited for different biological functions in all kingdoms of life (Iglesias et al., 2015; Cámara-Almirón et al., 2018). However, the amyloid structure entails an inherent risk that, without control, could lead to a succession of tragic events able to cross the species barrier, such as in the case of the transmissible spongiform encephalopathies (Zhou et al., 2012; Revilla-García et al., 2020; Sampson et al., 2020).

**TABLE 1:** Examples of interspecies interactions between amyloid proteins. List of exogenous amyloid proteins, that can be in the gut, and that interfere with the aggregation of unrelated human amyloid proteins.

Exogenous protein	Organism	Human protein	Interaction effect	References
FapC	*Pseudomonas*	α-synuclein	Accelerates	Christensen et al. (2019)
FapC	*Pseudomonas*	Amyloid-β peptide	Accelerates	Javed et al. (2020)
CsgA	*Escherichia coli*	α-synuclein	Accelerates	Sampson et al. (2020)
CsgA, CsgB	*Escherichia coli*	PAP	Accelerates	Hartman et al. (2013)
CsgA, CsgB	*Escherichia coli*	Amyloid-β peptide	Accelerates	Hartman et al. (2013)
CsgA, CsgB	*Escherichia coli*	IAPP	Accelerates	Hartman et al. (2013)
LPS endotoxin	Gram-negative bacteria	α-synuclein	Accelerates and induces distinct strains	Kim et al. (2016)
Sup35	*Saccharomyces cerevisiae*	Amyloid-β peptide	Accelerates	Koloteva-Levine et al. (2021)
β-parvalbumin	Fish	α-synuclein	Inhibits	Werner et al. (2018)
β-lactoglobulin	Bovine milk	α-synuclein	Accelerates	Vaneyck et al. (2021)
Lysozyme	Chicken egg white	α-synuclein	Accelerates	Vaneyck et al. (2021)

The long periods that the microorganisms stay in the body, due to infection or symbiosis, could facilitate amyloid cross-seeding events between host and microorganism (Otzen and Nielsen, 2008; Iglesias et al., 2015). Moreover, recent studies have demonstrated that bacterial amyloid structures can initiate the formation of amyloid aggregates upon interaction with human proteins (Table 1) (Otzen and Nielsen, 2008; Christensen et al., 2019; Revilla-García et al., 2020; Sampson et al., 2020; Vaneyck et al., 2021). Therefore, it is crucial to identify the microorganisms and precise mechanisms that can influence the aggregation of host proteins. This will help to understand their link with human disease and to design new therapeutic strategies, such as microbiome manipulation with probiotics or antibiotics.

## Bacteria Produce Amyloids to Deal With the Surrounding World

To understand how human amyloidogenesis could be affected by coexisting with a huge and diverse community of microorganisms, we first should learn about their potential to produce and manipulate amyloid fibrils. In an early work, Larsen and co-workers performed a systematic screening in several habitats (seawater, sludge, and drinking water) and, in all of them, they detected between 5% and 40% of amyloid-positive bacteria, demonstrating that amyloid-forming proteins are widespread in this Domain (Larsen et al., 2007). Later, sequential and structural analyses provided enough information to generate computational tools capable to screen, in whole proteomes, for amyloid-forming proteins and prion-like domains (PrLDs), with potential to propagate the amyloid conformation (Espinosa Angarica et al., 2014; Lancaster et al., 2014; Iglesias et al., 2015; Iglesias et al., 2021; Yuan and Hochschild, 2017; Harrison, 2019). These data show that prion-like proteins are conserved across multiple phyla (Harrison, 2019) and that at least 0.3% of all known bacteria genes encode for PrLDs. However, for certain species, especially pathogenic bacteria such as *Staphylococcus aureus*, *Enterococcus faecalis*, *Enterococcus faecium*, or *Staphylococcus epidermidis*, this percentage could be higher and achieve 18% (Espinosa Angarica et al., 2013; Iglesias et al., 2015; Yuan and Hochschild, 2017).

A more detailed analysis shows that bacteria functional amyloids are mainly extracellular (Blanco et al., 2012). This could reduce the potential intracellular toxicity decreasing the cost associated to control it. But more importantly, at this location amyloid-forming proteins can interact with the sounding environment and develop roles of sensing and adaptation. As a result, bacterial amyloid proteins tend to be associated with adhesion, biofilm formation, and invasion (Elliot et al., 2003; Gebbink et al., 2005; Barnhart and Chapman, 2006). Prokaryotes also use the amyloid conformation to regulate toxins activity by inactivating or storing them. An example of this is Microcin E492 (Mcc), a pore-forming bacteriocin produced by *Klebsiella pneumoniae*. When exported, the monomers and oligomers create cytotoxic pores that induce the lysis of neighbouring bacteria. On the contrary, the amyloid structures act as inactive reservoirs able to sense environmental changes and to identify the right moment to release the monomers (Shahnawaz and Soto, 2012).

Adhesins acquire macromolecular structures to bind external elements and to build biofilms, three-dimensional matrices involved in host colonization (Barnhart and Chapman, 2006; Larsen et al., 2008; Dueholm et al., 2010). Importantly, biofilm formation enhances bacteria resistance to antibiotics. This is a big problem that increases the risk of mortality and health economic costs (Matilla-Cuenca et al., 2021; Sikora and Zahra, 2021). These infections are mainly caused by opportunistic bacteria such as *Enterococcus faecium, Staphylococcus aureus, Klebsiellapneumoniae, Acinetobacter baumannii,* or *Pseudomonas aeruginosa* (Ma et al., 2019; Matilla-Cuenca et al., 2021). Between them, *S. aureus* is one of the most common causes of hospital-acquired bacteremia (Jensen et al., 1999). Remarkably, this specie is equipped with a diverse set of biofilm-forming proteins able to accomplish multiple functions (Kosolapova et al., 2020; Zaman and Andreasen, 2020; Miller et al., 2021). For example, Bap protein develops a dual role, sensing environmental changes and scaffolding biofilm structures in response (Valle et al., 2020).

The expression of functional amyloid-forming proteins entails risk and bacteria must equip themselves with security mechanisms: chaperones that protect from aggregation, spatial compartmentalization, and temporal control. A clear example of this are the extracellular curli fibers that help in cell-to-cell contacts for community behaviour and host colonization (Gophna et al., 2001). Curli extracellular matrix formation is the result of a coordinated action between several structural and scaffolding components. In *E. coli* these proteins are encoded by seven different genes (csg) divided in two different operons (csgBAC and csgDEFG) (Bhoite et al., 2019). Curli fibrils production follows a precise and specific process, the type VII secretion system also known as the nucleation-precipitation pathway (Desvaux et al., 2009; Bhoite et al., 2019). Another key element are the chaperones CsgC and CsgE that impede the amyloid assembly until the csg proteins are transported outside (Nenninger et al., 2011; Evans et al., 2015; Otzen and Riek, 2019).

## Microbiota and Amyloid Diseases

Amyloid diseases are characterised by the aggregation of proteins into amyloid fibrils and their deposition into plaques and intracellular inclusions (Guerreiro and Hardy, 2014; De Groot and Burgas, 2015; Walker et al., 2016). They are the consequence of genetic and environmental factors, together with aging (Pang et al., 2019). During the last 10 years, several works also pointed out that one of these factors could be an altered microbiota (Chen et al., 2021). In fact, microbiome composition also changes with environmental factors and over time (Finlay et al., 2019; Badal et al., 2020; Bosco and Noti, 2021).

Recently, several publications reported altered gut populations in patients with neurodegenerative diseases (Hill-Burns et al., 2017; Peterson, 2020). The gut and the brain are interconnected by a bidirectional axis. Indeed, the gut contains around 100 million neurons, more than the spinal cord or the peripheral nervous system (Uesaka et al., 2016). It has also been identified as the main entrance of prions into the central nervous system in diseases such as bovine spongiform encephalopathy and kuru (Kujala et al., 2011). And it is also the route that allows microbiota and their products (lipopolysaccharides, amyloids, and other metabolites) to bypass the circulatory system (Braniste et al., 2014; Friedland and Chapman, 2017). This, together with the fact that human amyloid proteins such as amyloid-β-peptide (Aβ) can be found in the peripheral circulation and in the cerebrospinal fluid (Tublin et al., 2019; Wang et al., 2021), can favour interspecies encounters and amyloid protein cross-seeding. It is also important to note that Aβ may be specifically designed to interact with microorganisms, acting as an antimicrobial peptide in host immune response. It can form fibrils that trap pathogens and disrupt their membranes (Kumar et al., 2016; Moir et al., 2018).

Recently, Chen et al. (2016) reported a very original study of how bacterial amyloid aggregates affect rat models of Parkinson’s disease. They studied rats with guts colonised by two *E. coli* strains just differentiated by encoding for curli proteins with different capacity to form amyloid aggregates. Those bacteria expressing the aggregation-prone variant grew in rats with increased alpha-synuclein accumulation and enhanced cerebral inflammation, thus linking the formation of bacteria amyloid with exacerbated neurodegenerative symptoms. Sampson and colleagues transferred different human microbiotas to mice; and observed greater motor impairment in those animals with intestinal microbes from Parkinson’s patients than in those with microorganisms obtained from healthy persons (Sampson et al., 2016). Instead, Harach and co-workers studied the microbiota of mice models of Alzheimer’s disease (Harach et al., 2017). Their results indicate that the overexpression of Aβ generates a mixture of microbes that when transferred into germ-free mice exacerbates the Alzheimer’s pathology. Overall, there is much evidence that microbiota can influence the development of human disease, but how it happens at the molecular level remains elusive.

## How Do Microbiota Produced Amyloids Affect Amyloidogenesis

Amyloid fibrils have the intrinsic potential to self-propagate their β-sheet structure and template it on other soluble molecules (Table 1) (Morales et al., 2010). This seeding has been also detected between bacterial and host amyloid proteins (Friedland, 2015; Chen et al., 2016; Eisenberg and Sawaya, 2017). For example, *Pseudomonas* FapC protein forms amyloid fibrils for biofilm scaffolding, but in the body, these fibrils can trigger Aβ aggregation and influence the development of neurodegenerative diseases (Javed et al., 2020). In addition, curli fibrils from different bacterial species can seed human proteins aggregation both *in vitro* and *in vivo* (Lundmark et al., 2005; Zhou et al., 2012). Intriguingly, seeding reactions with heterologous sequences (also called cross-sending) can lead to alternative amyloid strains, fibrils with different conformational properties, that can cause different clinical severities of the same neurodegenerative disease (Chaudhuri et al., 2019; Javed et al., 2020; Ivanova et al., 2021).

The process of amyloid seeding can be influenced by both structural conformation and sequence. On one hand, the cross-seeding is enhanced when more than 70% of the sequence is shared (Wright et al., 2005). On the other hand, there is an increasing number of examples where fibrils, from unrelated sequences, accelerate the aggregation of a protein target more efficiently than its own fibrils (Wright et al., 2005; Dubey et al., 2014; Koloteva-Levine et al., 2021). The common structure responsible for the conformation propagation is thought to be cross-β-sheet, however, in heterologous seeding, these interactions may vary depending on the proteins involved (Ivanova et al., 2021). Amyloid seeding is achieved when the addition of preformed fibrils, in the aggregation reaction, provides compatible nuclei (or seeds) from which new fibrils can grow exponentially. Without seeds, fibrils growth is delayed until the protein monomers achieve to self-assemble and build *de novo* nuclei, this is a critical phase that can last from minutes to days (Ivanova et al., 2021).

Despite that there are still many questions to be solved at a molecular level, different mechanisms have been proposed to explain the amyloid seeding between heterologous sequences. Two of the most accepted mechanisms are th*e template-assisted and the conformational selection and population shift. In both cases,* the heterologous amyloid fibrils provide an electrostatic environment and hydrophobic surfaces that favour the nucleation and growth of new aggregates (Ren et al., 2019; Koloteva-Levine et al., 2021; Subedi et al., 2022). At the *template-assisted* mechanism the protein that grows amyloid fibrils faster, or at least the one with more fibrils, seeds the molecules of the other amyloid protein. And at the *conformational selection and population shift* mechanism, both proteins have a similar number of seeds, and both types of amyloid fibrils adjust their conformations to bind each other and cross-seed (Ren et al., 2019; Ivanova et al., 2021; Subedi et al., 2022).

## Microbiome Manipulation as a Therapeutic Strategy

The link between gut dysbiosis and neurodegenerative diseases is inspiring new therapeutic strategies based on microbiota manipulation (Figure 1) (Peterson et al., 2015; Peterson, 2020). This can be achieved with dietary treatments such as probiotics or faecal transplantation. Probiotics consumption can increase the levels of fatty acids in the brain, supporting brain function, learning, memory, and neurogenesis (Strandwitz, 2018; Peterson, 2020). It also can decrease psychological stress, recover immune response, and improve anxiety in patients with chronic fatigue syndrome (Rao et al., 2009; Messaoudi et al., 2011). However, gut microbiota can also be manipulated with antibiotics. In 2016, Minter et al. (2016) showed that the administration of antibiotics, to mouse models of AD, can reduce gut microbial diversity and decrease amyloidosis and neuroinflammation.

In patients with Alzheimer’s disease, probiotics have anti-oxidant and anti-inflammatory effects that ultimately can cause cognitive recovery (Rao et al., 2009; Messaoudi et al., 2011; Bonfili et al., 2017; Kobayashi et al., 2017; Abraham et al., 2019; Deng et al., 2020). Recently, Govindarajan and co-workers studied for 12 weeks the effect of a probiotic milk containing *Lactobacillus acidophilus*, *Lactobacillus casei*, *Bifidobacterium bifidum*, and *Lactobacillus fermentum*. In this trial, the AD patients presented several improvements including cognitive performance but without a decrease in inflammation or oxidative stress (Leblhuber et al., 2018). Studies on mice models of AD support that transplantation of faecal microbiota from healthy people to patients can improve the composition of the intestinal microbiota and alleviate the disease symptoms (Rogers et al., 2016). Also in mice models, the administration of certain bacteria strains, such as *Lactobacillus plantarum* or *Bifidobacterium breve A1*, decreases Aβ deposition and improves the behavioural deficits (Bonfili et al., 2017; Kobayashi et al., 2017; Lee et al., 2018).

Finally, there are also interesting strategies that focus on interfering with the aggregation-prone protein or even use amyloid peptides as therapeutic agents. Recently, Henning-Knechtel et al. (2020), (Abdelrahman et al., 2020), combined the sequence of different amyloid proteins (Prion Protein, Aβ, and NCAM1 glycoprotein), to design cell-penetrating peptide inhibitors of Aβ fibrillation (Tjernberg et al., 1996; Soto et al., 1998; Lowe et al., 2001; Österlund et al., 2019; Abdelrahman et al., 2020). These peptides also prevent the formation of toxic oligomers and can bind both extra- and intracellular Aβ. Therefore, not just microbiota but also its metabolites (such as their amyloid proteins) can be targeted by therapeutic strategies.

## Discussion

With a regular and strong structure, amyloid fibrils are produced to develop functional roles in all kingdoms of life. However, their propagation capacity also entails a risk that without control can have fatal consequences. Relevantly, our bodies contain around 2 Kg of microorganisms (Pagliari et al., 2015) encoding in their genes at least 0.3% of potential prion-like sequences (Iglesias et al., 2015). Moreover, these microbes can reside in our bodies for very long periods. All these facts support that interspecies cross-sending may happen more often than previously expected and could be the origin of several health disturbances (Figure 1).

The study of the microbiome is starting to reveal information about our relationship with microorganisms (NIH Human Microbiome Project—HMRGD). However, there are still lots of unsolved questions about how microbiota and their metabolites influence human health and disease. This is a promising area with a broad range of possible strategies that can be based not only on microbiota manipulation but also on interfering with their metabolites.
